# Conditions of Admission to the Register

**Published:** 1921-04-23

**Authors:** 


### NURSE REGISTRATION IN IRELAND.

### Conditions of Admission to the Register.

The rules recently issued by the General Nursing Council for Ireland set out definitely under what conditions nurses already trained and in practice (existing nurses) and nurses who are in process of being trained (interim nurses) can obtain admission to the General Part of the Register, or to either of its four Supplementary Parts—(1) for male nurses; (2) for mental nurses; (3) for sick children's nurses; (4) for fever nurses.

### Existing Nurses.

In many respects the same conditions govern admission to all parts of the Register. We give below those which relate to admission to the General Register, specifying in what particulars, if any, variations occur in the rules for admission to either of the Supplementary Parts.

Date of Application.—Existing nurses must make application within two years from the date on which this part of the Rules came into operation—*i.e.*, before March 31, 1923.

Form of Application.—The printed form on which application must be made can be obtained free on application to the Registrar, 33 St. Stephen's Green, Dublin.

Fee.—The fee for admission to the General Part of the Register, or to any one of the Supplementary Parts, is £1 1s. Applicants who have been admitted to the General Register, or have paid the fee for admission to it, can be admitted to any of the Supplementary Parts at a fee of 10s. 6d.

Evidence as to Character.—All applicants must satisfy the Council that they are of good moral character, and to this end must furnish the names of two references, matrons or medical practitioners under whom they have worked, or other persons (not relatives) holding responsible positions, who have known them personally for at least three years, and can furnish a certificate of good character for at least the three years immediately preceding date of application.

Age of Applicant.—Candidates for admission to the Register must furnish certificate of birth or baptism, or a statutory declaration to show that they are not under twenty-one years of age.

Marriage Certificate.—This must be produced by female married or widowed applicants.

Professional Qualification.—(1) *General Part of the Register*.—There are three classes of nurse who can claim admission. 1. The nurses who show that they have carried out not less than three years' approved training in a general hospital or Poor-Law infirmary. 2. Nurses who have carried out not less than one year's approved training in a general hospital or Poor-Law infirmary, and been *bona-fide* engaged in practice as nurses for at least two years subsequent to the period of training. 3. Nurses who have been employed with the approval of the Local Government Board for Ireland as "qualified" nurses for at least three years.

(2) *Supplementary Part of Register for Male Nurses.*—There are two classes. 1. Male nurses with three years' approved training in a hospital or institution, or in the service of the Admiralty, the Army Council, or the Air Council. 2. Male nurses with not less than one year's approved training in such hospital or institution or in one of the above services, with at least two years' subsequent *bona-fide* practice in nursing.

(3) *Supplementary Part of Register for Mental Nurses.*—The admission of four classes of mental nurses is provided for. 1. Nurses who can show the certificate of the Medico-Psychological Association certifying that they have completed their training and passed the examination prior to November 1919. 2. Nurses with not less than three years' approved training in a hospital or institution for the treatment of mental diseases. 3. Nurses with not less than two years' approved training in such hospital or institution, followed by one year's *bona-fide* practice in the nursing and care of persons suffering from mental diseases. 4. Applicants experienced only in the nursing and care of the feeble-minded and mentally defective, provided they have been engaged for three years prior to November 1919 in such nursing, and have been employed for at least two years of that period as nurses in an institution for mental defectives.

(4) *Supplementary Part of Register for Sick Children's Nurses.*—The admission of two classes of nurses is provided for. 1. Nurses with three years' approved training in a hospital for sick children. 2. Nurses with not less than two years in such an institution, followed by at least one year's *bona-fide* practice in the nursing and care of the sick.

(5) *Supplementary Part of Register for Fever Nurses.*—The admission to the Register of three classes of nurses is provided for. 1. Nurses with not less than three years' approved training in a hospital for infectious diseases. 2. Nurses with not less than two years' approved training in such a hospital, followed by at least one year's *bona-fide* practice in the nursing of the sick. 3. Nurses with not less than three years' training in a general hospital or Poor-Law infirmary, with at least one year's training in a hospital for infectious diseases.

### Interim Nurses

Nurses who had not completed their training by November 1, 1919, the date of the passing of the Nurses' Registration Act, are subject to special regulations. These may be briefly summarised as designed to bring all these applicants under the regulations laid down for the first-named class of nurses in each part of the Register.

*General Trained Nurses* to have had three years' satisfactory training in a general hospital or Poor-Law infirmary, approved by the Council.

*Male Nurses* to have had three years in a hospital or approved institution, or in the service of the Admiralty, the Army Council, or the Air Council.

*Mental Nurses* to have had three years' training in approved institutions, or possess the certificate of the Medico-Psychological Association.

*Sick Children's Nurses* to have had three years' training in an approved general hospital for sick children.

*Fever Nurses* to have had three years' training in an approved hospital for infectious diseases.

The regulations are tightened up by the proviso in all cases that the institution in which the Interim Nurse has been trained must be one approved by the Council. The period of training in all cases must terminate on a date subsequent to November 1, 1919, but prior to the date on which the rules to be made by the Council for admission of nurses by examination become operative.

